# Drug Headaches

**Published:** 1904-07

**Authors:** 


### DRUG HEADACHES.

*American Medicine,* referring to the not uncommon practice of self-medication by advertised preparations containing soporific drugs, says that not only are gastric disturbances produced by the drugs taken, but often headaches occur which are characteristic. "In certain individuals," says the editor, "these effects are produced only after excessive and prolonged use of the drug; in others, such as nervous and neurasthenic persons, small doses at times cause grave disturbance. In these patients it is often manifested by severe and persistent headache. Although very frequent, drug headaches are often unperceived or ascribed to some indiscretion. It is important that the physician does not overlook it. * * * In all patients suffering from a rebellious headache careful inquiry should be instituted to determine if any drug has been taken which might produce it."

